# The role of basic residues in the adsorption of blood proteins onto the graphene surface

**DOI:** 10.1038/srep10873

**Published:** 2015-06-02

**Authors:** Zonglin Gu, Zaixing Yang, Lingle Wang, Hong Zhou, Camilo A. Jimenez-Cruz, Ruhong Zhou

**Affiliations:** 1 Institute of Quantitative Biology and Medicine, SRMP and RAD-X, Collaborative Innovation Center of Radiation Medicine of Jiangsu Higher Education Institutions, Soochow University, Suzhou 215123, China; 2 IBM Thomas J. Watson Research Center, Yorktown Heights, NY 10598, USA; 3 Department of Chemistry, Columbia University, New York, NY 10027, USA

## Abstract

With its many unique properties, graphene has shown great potential in various biomedical applications, while its biocompatibility has also attracted growing concerns. Previous studies have shown that the formation of protein-graphene corona could effectively reduce its cytotoxicity; however, the underlying molecular mechanism remains not well-understood. Herein, we use extensive molecular dynamics simulations to demonstrate that blood proteins such as bovine fibrinogen (BFG) can absorb onto the graphene surface quickly and tightly to form a corona complex. Aromatic residues contributed significantly during this adsorption process due to the strong π−π stacking interactions between their aromatic rings and the graphene *sp^2^*-carbons. Somewhat surprisingly, basic residues like arginine, also played an equally or even stronger role during this process. The strong dispersion interactions between the sidechains of these solvent-exposed basic residues and the graphene surface provide the driving force for a tight binding of these basic residues. To the best of our knowledge, this is the first study with blood proteins to show that, in addition to the aromatic residues, the basic residues also play an important role in the formation of protein-graphene corona complexes.

A variety of carbon-based nanomaterials (CBNs), such as C_60_, carbon nanotubes (CNT) and graphene, have attracted considerable attention in many fields ever since their discovery^1^,^2^,^3^. Of particular interest are applications in the field of biomedicine^4^,^5^,^6^,^7^, where the CBNs enable new and exciting technologies. Different from traditional macroscopic materials, CBNs have many unique physicochemical properties, such as extensively high specific surface area^8^, size and dimensional effects^9^, vast structural amenability, in addition to their excellent mechanical and electrical properties^10^,^11^. Therefore, they have been proposed for usages in drug delivery platforms, diagnostic agencies and therapeutic nanodrugs^12^,^13^,^14^. As an example, because of their excellent biopersistence, smooth and contiguous topography, graphene and its derivative graphene oxide (GO) play a unique role in the foreign-body-induced carcinogenesis and tumor progression^15^. Some recent studies have also shown that graphene and GO have strong antibacterial capabilities, making them potential candidates for the next generation “green” antibiotics^16^,^17^,^18^,^19^. Moreover, because of the ultra-high *in vivo* uptake in tumor cells (in mice) of graphene^20^, this CBN can be potentially used as an effective agent for photothermal ablation of tumors^21^. Similarly, our recent studies have shown that, Gd@C_82_(OH)_22_, a C_82_ ramification containing a caged heavy metal element, can bind and inhibit the activity of metalloproteinases-9 (MMP-9) and metalloproteinases-2 (MMP-2), which can in turn effectively suppress tumor angiogenesis, invasion and metastasis^22^. All these evidences are depicting the soon-to-come promising usage of CBNs in biomedicines.

Despite these advantages, the biosafety/cytotoxicity of CBNs, like one of the two sides of a coin, also has attracted growing concerns of a broad scientific community^23^,^24^,^25^. The cytotoxicity of CBNs has been reported from different aspects. Many recent *in vitro* experimental and theoretical studies have shown that both, the bare graphene and GO, can directly kill bacteria and mammalian cells by penetrating into cell membranes and extracting large amounts of phospholipid molecules from the lipid bilayer^16^. Therefore, while GO has the potential to be designed as a new generation of “green” anti-bacterial drug, it may also potentially make a wrong execution to the healthy cells (particularly when alone) and cause unwanted cytotoxicity. This cytotoxicity has also been associated with other nanomaterials and mechanisms, as in our recent studies showing that certain sized single-walled CNT can easily win the competitive binding to the protein receptor over its native peptide ligand^26^, indicating a potential toxicity to biological signal-conduction process. Many other studies also demonstrate that GO exhibits non-negligible cytotoxicity by increasing the activity of some unwanted biological enzymes (such as caspase 3)^27^, or by generating a large number of reactive oxygen species (ROS)^28^,^29^. Therefore, it is very important to systematically study the molecular origins of CBNs’ cytotoxicity, shedding light on the new design strategies to mitigate their cytotoxicity on large-scale biomedical applications.

In therapeutic or diagnostic applications of CBNs, the blood circulatory systems will most likely be the first organ to interact with these materials. Upon entry of the CBN into the microenvironment of bloodstream, massive biomolecules (such as proteins and lipids) quickly adsorb onto CBNs, forming the so-called corona complex^30^. Formation of a corona complex largely reduces the surface free energy and alters physicochemical properties of the nanomaterial, changing in this way their function and surface morphology. More and more studies have shown that the induced cytotoxicity of the CBN is largely relieved when the CBNs are coated with fetal bovine serum (FBS) proteins or other similar proteins^30^,^31^,^32^. For example, our previous studies have illustrated that four types of the most abundant human serum proteins, BFG, gamma globulin (Ig), Transferrin (Tf) and Bovine serum albumin (BSA), can competitively adsorb onto the surface of CNTs to form protein-coated CNTs corona, and coating with these different proteins can fundamentally alter CNTs’ cellular interaction pathways and reduce their cytotoxicity^30^. This effect was particularly strong in the BFG-coated CNTs^30^. These findings suggest a feasible direction to mitigate the cytotoxicity of CBNs and uncover the essence of interactions between CNTs and human serum proteins. However, in view of the huge differences in the physicochemical properties (i.e., shape, dimension and charge) of various CBNs, many fundamental questions about how the different types of CBNs interact with human serum proteins remain unclear, such as the detailed molecular mechanisms of the adsorption process and the key factors controlling the adsorption process.

In this study, all-atom molecular dynamics (MD) simulations were performed to examine the adsorption process of BFG and BSA onto the graphene surface. Both the largest (BFG) and smallest (BSA) protein among the four blood proteins were chosen for this study, with the BFG being the major target protein mainly because of its unique and special 3D topological structure with long-intertwining flexible *α*-helix chains. As shown in our previous study^30^, different from the other three blood proteins, BFG’s long and flexible *α*-helix tails act as tentacles, which can wrap along the CNTs and thus expose more inner residues to the CNT surfaces (and resulting in particularly strong effects in reducing the cytotoxicity of CNTs)^30^. On the other hand, BSA is a more stable globular protein. Graphene nanosheets were chosen because improved manufacturing methodologies have led to an increase of attention on these nanomaterials in the biomedical field^33^,^34^,^35^. As one of the most abundant human serum proteins, BFG interactions with the graphene sheets are of remarkable importance once the nanomaterials enter the bloodstream. BFG, as the principal protein of blood clotting, has several important physiological functions, including to bridge platelets by binding to their GpIIb/IIIa surface membrane proteins^36^, or perhaps more importantly, to act as the fibrin precursor. For this reason, overexpression of fibrinogen is believed to be an independent risk factor for inflammation and cardiovascular diseases because of its ability to promote thrombus formation^37^. A detailed understanding of the adsorption process of BFG onto the graphene surface and the underlying mechanisms is then crucial to unveiling the molecular origins of the cytotoxicity of graphene, providing insight to new strategies to enhance biocompatibility of CBNs.

## Models and Methods

The coordinates of the graphene nanosheets used in the simulations were generated using the nanotube builder plugin of the VMD software^38^. Similar to the previous studies^39^, the carbon atoms of the graphene were modeled as uncharged Lenard-Jones particles with a cross-section of *σ*_*cc*_ = 0.34 nm and a potential well depth of *ε*_*cc*_ = 0.3598 kJ mol^−1^. Carbon-carbon bond lengths and bond angles were maintained by harmonic potentials with spring constants of 392,460 kJ mol^−1^ nm^−2^ and 527 kJ mol^−1^rad^−2^ respectively.

**Figure 1:**
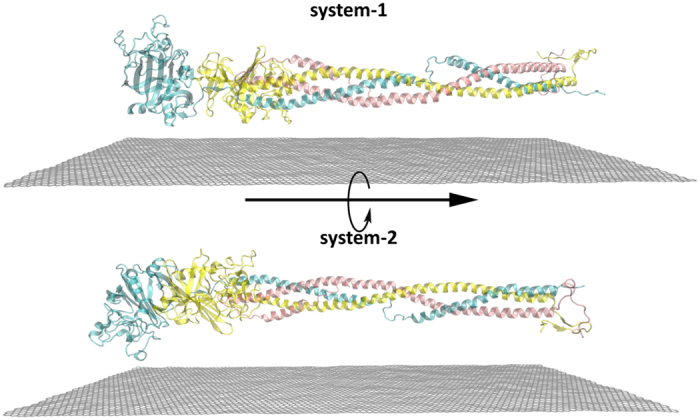
Two different initial configurations used in the adsorbing simulations of BFG onto the surface of graphene nanosheet. The *α*-chian, *β*-chain and *γ*-chain are shown in pink, yellow, and cyan, respectively. Graphene is shown with gray-flat-sheet. System-2 is derived from system-1, by rotating the protein by 180° along the inset axis. For clarity, water molecules are not shown.

The BFG crystal structure was obtained from RCSB Protein Data Bank (with PDB ID: 1DEQ)^40^, which is the main target protein in this study (with all results refer to BFG unless otherwise explicitly stated for BSA). As mentioned above, the full BFG protein complex is a hexamer, with two homologous halves linked by disulfide bonds. Each half of the hexamer consists of three different long-intertwining chains (*α β* and *γ*) (see Fig. 1 for more details; the setup of BSA is shown in Supporting Information). The length of a full BFG hexamer is about 450 Å. Similar to our previous study^30^, in order to reduce the computational cost, only one half of the BFG hexamer was modeled in our simulations. To rule out the dependence of our findings on the initial configurations of the simulations, two different system setups were used in the simulations, which differ by the relative orientations of the proteins with respect to the graphene surface. As shown in Fig .1, system-2 is derived from system-1 by rotating the protein along the axis parallel to the three long chains by 180°.

System-1 consists of 612,464 atoms (with box size of 31.91 nm × 12.96 nm × 14.96 nm and graphene size of 25.66 nm × 9.90 nm) while system-2 consists of 621,358 atoms (with box size of 32.06 nm × 13.02 nm × 15.03 nm and graphene size of 25.66 nm × 9.90 nm). The distance between any heavy atom of the BFG and the graphene surface is greater than 1.0 nm in the initial configurations of the simulations. The solvated systems were then simulated with MD, which is widely used in the studies of both biomolecules^41^,^42^,^43^,^44^,^45^,^46^,^47^ and nanomaterials^24^,^39^,^48^,^49^,^50^,^51^. Here, the MD simulations were performed using the software package GROMACS (version 4.6.4)^52^ with CHARMM 27 force field^53^. The TIP3P water model was used for the water molecules^54^. The simulation temperature was maintained at 310 K using *v*-rescale thermostat^55^ and the pressure was kept at 1 atm using Berendsen’s algorithm^56^. Periodic boundary conditions were applied in all directions. The long-range electrostatic interactions were treated with PME method^57^, and the van der Waals (vdW) interactions were calculated with a cutoff distance of 1.2 nm. All solute bonds were constrained to their equilibrium values employing the LINCS algorithm^58^ and water geometry was constrained employing the SETTLE^59^ algorithm. During the production runs, a time step of 2.0 fs was used, and coordinates were collected every 20 ps. Three independent simulations of 200 ns were performed for each of the two systems. The total aggregated simulation time is longer than 1.2 μs.

## Results and discussions

The adsorption process of BFG onto the graphene surface was interrogated by calculating the contact surface areas and the contact number between heavy atoms of the two molecules. As a measure to monitor the time evolution of the overall structure of BFG during the absorption process, the root mean square deviation (RMSD) of the heavy atoms of BFG compared with the crystal structure and the fraction of native contacts Q were also calculated. Here, an atom-atom pair contact is defined when the distance between any heavy atom of BFG and any carbon atom of graphene is smaller than 6.0 Å. Two residues are considered to have a native contact if the distance between any heavy atom of the *i*th residue (the first residue) and any heavy atom of another *j*th residue (*j* - *i* > 3) (the second residue) is shorter than 6.0 Å in the crystal structure of the protein. By definition, for the fully folded state, *Q* = 1, with decreasing Q as the instantaneous 3D structure of the protein resembles less the crystal structure.

**Figure 2:**
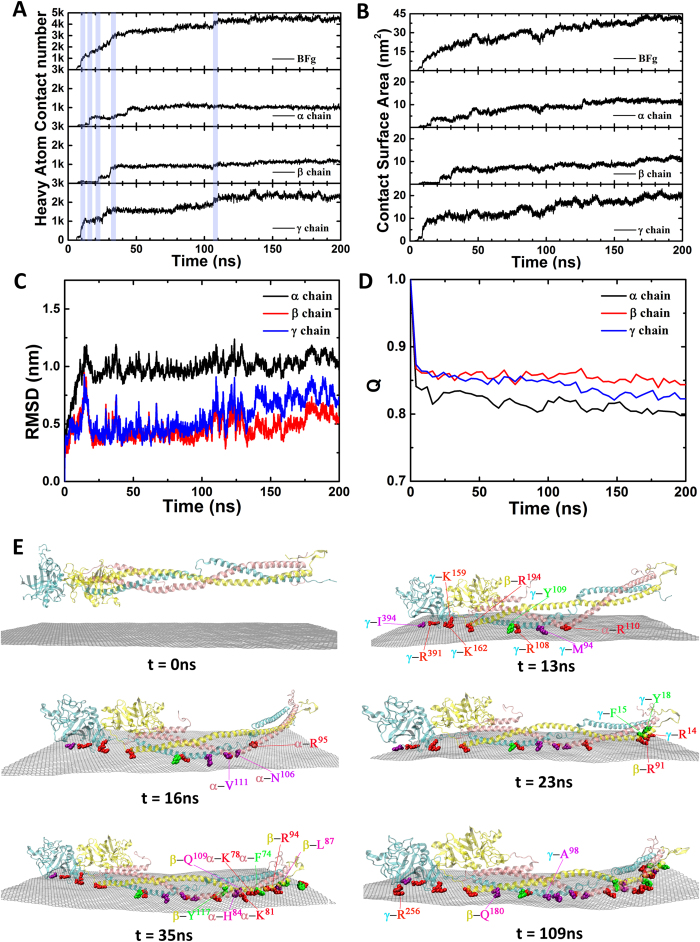
A representative trajectory of system-1 shown the adsorbing process of BFG onto the surface of graphene. A and B from top to bottom: the heavy atom contact number and contact surface area between graphene and BFG, and decompstion of these quantities into its branced chains (*α*-chian, *β*-chain and *γ*-chain), respectively. C and D: RMSDs and Q values of the *α*-chian, the *β*-chain and the *γ*-chain as a function of simulation time. (**E**) Some important intermediate states, at t = 0, 13, 16, 23, 35 and 109 ns, near several important sudden jumps in the total heavy atom contact number. Some important residues in the adsorbing process are highlighted with vdW ball, among which the basic amino acids are colored in red, the aromatic amino acids are colored in green and the hydrophobic amino acids are colored in purple.

The heavy atom contact number between BFG and graphene as a function of simulation time is shown in Fig. 2A (top panel). During the first ~35 ns of the simulation, the heavy atom contact number increased very quickly from 0 to ~3300. The contact number continued to increase a slower rate between 35 ns to 150 ns (increased from ~3300 to ~4490), and then fluctuated around ~4490 after 150 ns. It is interesting to note that there are several steep increases (such as at t = 13, 16, 23, 35 and 109 ns) in the curve of the heavy atom contact number. This kind of successive-wave-propulsion adsorbing pattern is mainly due to the asynchronous adsorption of various parts of BFG. The total heavy atom contact number between BFG and graphene was decomposed into its three branched chains (Fig. 2A bottom panels), showing that the steep increases in the total heavy atom contact number corresponds to a jump in the heavy atom contact number of one of its branched chains. (Similar trends were also observed for system-2 (Fig. S1)). For example, a jump at t = 13, 16 and 23 ns in the total heavy atom contact number corresponds to a jump in the contact number of the *γ*-chain (cyan), the *α*-chain (pink) and the *β*-chain (yellow) (see Fig. 2A for more details), respectively. Very similar trend was also observed for the contact surface areas between graphene and BFG (see Fig. 2B).

To monitor the structure change of BFG during the adsorption process, the heavy atom RMSD and the fraction of native contact *Q* as function of simulation time for the protein and its three branched chains are shown in Fig. 2C,D. After about 50 ns equilibration time, the RMSDs were about 1.05, 0.54 and 0.73 nm for the *γ*-chain, the *α*-chain and the *β*-chain, respectively (Fig. 2C), with the average *Q* values about 0.80, 0.85 and 0.83 (Fig. 2D), respectively. During the rest of the simulations, the RMSDs and *Q* fluctuated slightly around these values, indicating that the overall structure of BFG and its three branched chains were well maintained during the adsorption process.

To get more detailed molecular picture of the adsorption process, some important intermediate states leading to the sudden jumps in the heavy atom contact number between BFG and graphene were carefully examined. As shown in Fig. 2E, during the first 13 ns, the head of the *γ*-chain was firstly adsorbed onto the graphene while the other parts of protein remained detached from the graphene. During this adsorbing process, Ile-394, Arg-391, Lys-159, Lys-162, Tyr-109, Arg-108 and Met-94 in the *γ*-chain, Arg-194 in the *β*-chain, and Arg-110 in the *α*-chain played a very important role. After 16 ns, the adsorption gradually spread from the head to the tail, and Val-111, Asn-106 and Arg-95 in the *α*-chain started to bind to the graphene surface, a critical step anchoring *α*-chain to the graphene. At about t = 23 ns, Arg-91 in the *β*-chain, Arg-14, Phe-15 and Tyr-18 in the *γ*-chain began to approach the graphene surface, driving the tail of the protein to bind to the graphene surface. After 35 ns, the adsorption of the protein was fully under way, and an increasing number of detached residues located in the tail were adsorbed onto the graphene surface, such as His-84, Lys-81, Lys-78 and Phe-74 in the *α*-chain, and Tyr-117, Gln-109, Arg-94 and Leu-87 in the *β*-chain. At t = 109 ns, the small jump in the heavy atom contact number was mainly due to the adsorption of residues Gln-180 in the *β*-chain, and Arg-256 and Ala-98 in the *γ*-chain. These analyses clearly show that each round of increase in the heavy atom contact number between BFG and graphene was driven by the asynchronous adsorption of several key residues. Among those key residues identified in the adsorption process, the aromatic amino acids, such as Tryptophan, Tyrosine and Phenylalanine, take a significant proportion. To our surprise, the basic amino acids such as Arginine and Lysine, take even a larger percentage in key residues as compared to the aromatic amino acids, which will be explained in detail in the following sections.

**Figure 3:**
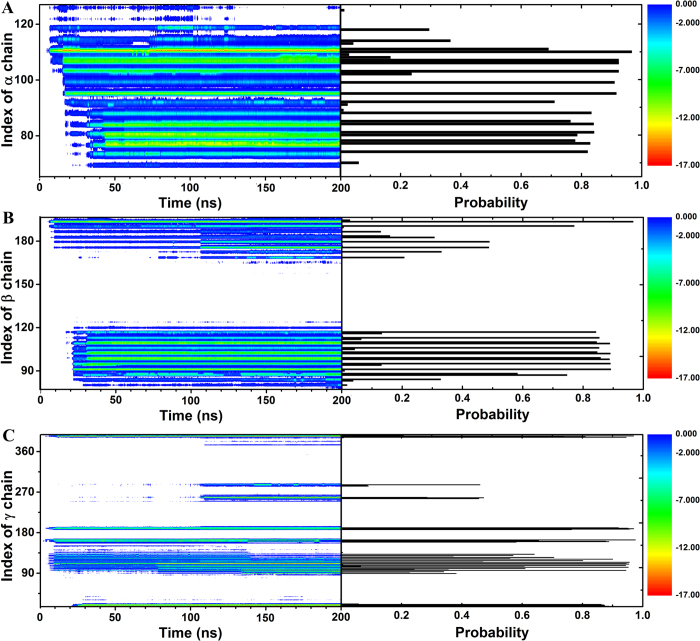
Time evloution of the vdW interaction energy (kcal/mol) between each residue and the graphene (the first column) and their contact probility (the second column) for the *α*-chain (A), the *β*-chain (B) and the *γ*-chain (C).

To quantify the contribution of each residue to the adsorbing process, we computed the time evolution of the vdW (or dispersion) energy between each residue and graphene (first column in Fig. 3) and their contact probabilities over the trajectory (second column in Fig. 3). As shown in the first column of Fig. 3A–C, the decrease in the vdW energy often occurred cooperatively for several residues during the adsorbing process. For example, at about t = 10 ns, the vdW energy started to decrease substantially for residues Ile-394, Arg-391, Lys-159, Lys-162, Arg-108 and Met-94 in the *γ*-chain, Arg-194 in the *β*-chain, and Arg-110 in the *α*-chain, especially for residues Arg-391 (decreased by 16.65 ± 0.92 kcal/mol) and Arg-108 (decreased by 13.27 ± 1.44 kcal/mol) in the *γ*-chain and Arg-110 (decreased by 14.74 ± 0.81 kcal/mol) in the *α*-chain. Later on, at about t = 16 ns, another round of moderate decrease in the vdW energy occurred for residues Val-111, Asn-106 and Arg-95 in the *α*-chain, with the largest decrease for Arg-95 by 10.77 ± 0.82 kcal/mol. Then, at about t = 23 ns, a new round of even more dramatic decrease in the vdW interaction energy occurred for residues Arg-91 in the *β*-chain, Arg-14, Phe-15 and Tyr-18 in the *γ*-chain with even the smallest decrease for Arg-91 in the *β*-chain by 12.95 ± 0.88 kcal/mol. The magnitude of the decrease in the vdW energy for each residue indicates how strongly it binds to the graphene surface. The more dramatic the decrease of the vdW energy, the tighter the binding of the residue to the graphene. Therefore, Arg-110 in the *α*-chain, Arg-91 in the *β*-chain, Arg-14, Phe-15, Tyr-18, Arg-391 and Arg-108 in the *γ*-chain played a very critical role in the binding of BFG to the graphene surface.

Not too surprisingly, the contact probability of each residue resembles that of the vdW interaction energy change profile (see Fig. 3 for more details): the more dramatic the decrease in the vdW energy for each residue is, the larger its contact probability. We then filtered out those residues whose contact probability with graphene is greater than say 0.6 (other values also tried, and similar trends were observed), and Arg-110 in the *α*-chain, Arg-91 in the *β*-chain, Arg-14, Phe-15, Tyr-18, Arg-391 and Arg-108 in the *γ*-chain stand out. This analysis further supports the above conclusion that these residues played an important role in the adsorption process.

**Figure 4:**
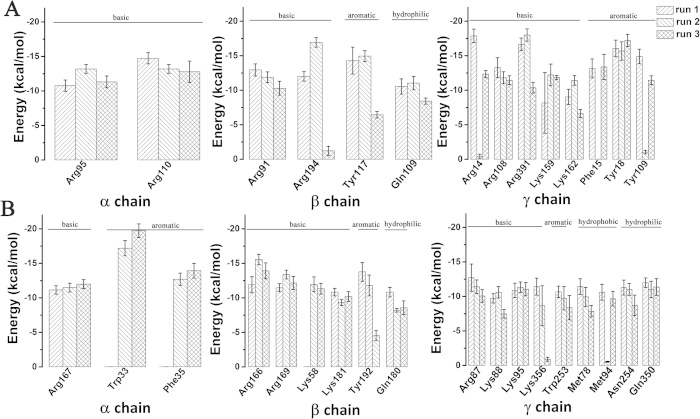
All the tagged key residues whose average vdW interaction energy with graphene is ≤ − 10 kcal/mol in at least two of the three simulations for system-1 (A) and system-2 (B).

The above analyses of the vdW interaction energy and the contact probability with graphene have identified several key residues in BFG during the adsorption process. To further validate these findings, we also tagged those residues whose interaction energy with the graphene is ≤ − 10 kcal/mol in at least two of the three simulations. The residues matching this criterion are shown in Fig. 4. They are Arg-95 and Arg-110 in the *α*-chain, Arg-91, Gln-109, Tyr-117 and Arg-194 in the *β*-chain and Arg-14, Phe-15, Tyr-18, Arg-108, Tyr-109, Lys-159, Lys-162 and Arg-391 in the *γ*-chain. These residues are within the list of the aforementioned key residues, confirming the important role they played in the adsorption process.

We expected the aromatic residues to drive the adsorption process, due to the strong π-π stacking interactions with graphene. Surprisingly, an overwhelming proportion (9/14) of the identified key residues are basic in nature (arginine and lysine) (see Fig. S2 for their distribution on the protein – they are mostly random in space). Simulations of system-2 also yielded similar results with the same population of the basic residues and aromatic residues identified as key for the process (see Fig. 4B). Additional simulations with the smaller protein BSA also show very similar results (more later; see Fig. S3). This seems to be at odds with our intuition, since the basic residues are expected to be favorable in water rather than paving onto the hydrophobic surface of graphene.

To clarify this puzzle, we carefully studied the detailed dynamics of some representative aromatic and basic residues in the adsorption process. We chose Arg-391 because it was found to play a very important role in the early adsorption of the head of protein. Similarly, we studied Tyr-18 since it was identified to be essential to anchor the protein tail onto the graphene surface. We monitored the time evolution of the heavy atom contact number between the side-chain of these residues and the graphene, and also computed the number of water molecules in the first solvation shell (FSS) of their side chains. Here, FSS is defined as the region within 5 Å to any heavy atom (non-hydrogen atom) of side chain.

**Figure 5:**
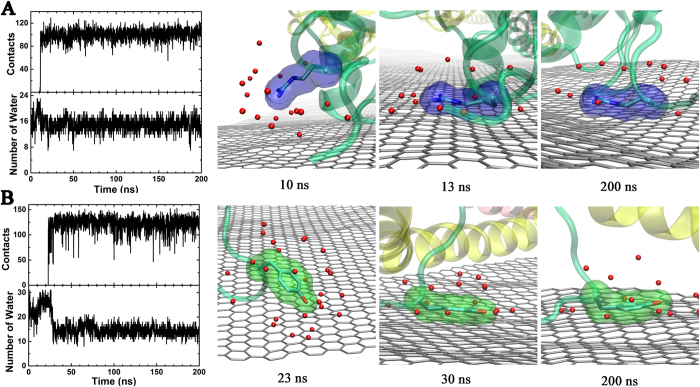
The heavy atom contact number between target residues and graphene (top panel in the first graph), the number of water molecules in FSS of target residue (bottom panel in the first graph), and several representative snapshots from the trajectory. (**A**) for Arg-391 and (**B**) for Tyr-18. The *α*-chian, *β*-chain and *γ*-chain are shown with pink, yellow, and cyan newcartoon, respectively, and the graphene are shown with gray-flat-sheet. The red spheres represent the water oxygen atoms in FSS of target residue sidechain.

The time evolution of the heavy atom contact number and the number of water molecules in the FFS for Arg-391 and Tyr-18 are shown in Fig 5. For Arg-391, the side chain (and especially the positive charged guanidinium tail) was fully solvated by ~18 water molecules (see the snapshot at t = 10 ns in Fig. 5A) during the first ~10 ns of the simulation before it started to approach the graphene surface. Then, during a very short time interval from ~10 to ~13 ns, it was quickly and fully adsorbed onto the graphene surface. During this process, the heavy atom contact number between the side chain and graphene abruptly increased from 0 to ~103, and the number of water molecules in FSS of Arg-391 side chain only slightly decreased from ~18 to ~15 (see Fig. 5). One snapshot of the trajectory at t = 13 ns where Arg-391 just got adsorbed on the graphene surface is shown in Fig. 5. We observed that both the long hydrophobic aliphatic chain and the positive charged guanidinium tail were fully paved onto the graphene surface and the water molecules in the interfacial region between the side chain and graphene were totally squeezed out. The other side of the side chain (opposite face towards the graphene) was fully solvated by water though. From then on, Arg-391 stayed in that conformation during the rest of the simulation, and the heavy atom contact number and the number of water molecules in FSS also kept nearly constant. The relatively small decrease in the number of water molecules in the FSS implies that the loss of the electrostatic component of solvation free energy of the positive charged guanidinium group is probably very small, which in turn is compensated by the favorable vdW interactions between the side chain and the graphene. Therefore, the binding of residue Arg-391 onto the graphene is mainly due to the strong vdW interactions (dispersion interactions) between its long and relatively planar sidechain (guanidinium groups) and graphene. This favorable Arg-graphene interaction is also consistent with Arg’s favorable interaction with GO -- a very recent study by Stauffer *et al.*^60^ on single amino acids’ binding with GO shows that Arg displays a slightly stronger interaction with GO than Trp -- although electrostatic interactions do play a role there due to oxidation of the graphene sheet. Similarly, another very recent study with single amino acids have shown that indeed basic residues such as Arg can have similar binding strengths as aromatic residues such as Trp on CNTs^61^. Therefore, these recent results from single amino acids also support our current findings with large proteins.

Considering the potentially favorable vdW interactions between Leu/Ile and graphene when they form close contact, it seems reasonable to expect that Leu/Ile should also play an equally or more important role than Arg/Lys in the adsorption of protein onto the graphene surface as they do not need to pay the desolvation penalty of the charged groups as Arg/Lys do. However, this argument ignores two important differences between Leu/Ile and Arg/Lys. First, the side chains of Arg/Lys are longer than Leu/Ile, making them more accessible to the graphene surface. Second, and more importantly, while Arg/Lys are usually exposed to solvent in the native structure of the protein, Leu/Ile are highly packed in the hydrophobic environment of the interior of the protein. Therefore, in contrary to Arg/Lys where the favorable vdW interactions between Arg/Lys and graphene compensate the relatively small desolvation penalty (water molecules in FSS only decreases from ~18 to ~15), the vdW interactions between Leu/Ile and graphene are not strong enough to compensate the loss of favorable interaction between Leu/Ile and its surroundings residues in the native structure of the protein. Consequently, Leu/Ile were not observed to have played as critical a role during this adsorption process.

For Tyr-18 in the *γ*-chain, it was far away from graphene and fully solvated by water during the first 23 ns of the simulation (Fig. 5B). Then, during a very short period from 23 ns to 30 ns, the heavy atom contact number between the side chain of Tyr-18 and graphene jumped from 0 to ~135, and the number of water molecules in FSS also dramatically decreased from ~28 to ~13. Two snapshots of the trajectory, one where Tyr-18 was far away from the graphene (at t = 13 ns), and one where it was adsorbed onto the graphene (t = 30 ns), are also shown in Fig. 5. Between 23 ns and 30 ns, driving by the strong π−π stacking interaction, the side chain of Tyr-18 kept rotating around and gradually packed its aromatic ring towards the graphene surface. The water molecules between the aromatic ring and graphene surface was pushed out during this process. After 30 ns, the heavy atom contact number and the number of water molecules in FSS of the sidechain of Tyr-18 kept relatively constant and the aromatic ring of Tyr-18 stayed stacked onto the graphene surface during the rest of the simulation. Therefore, the π−π stacking interactions dominated the whole adsorption process of Tyr-18 onto the graphene surface, consistent with previous experimental and theoretical studies reporting the importance of the π-π stacking interactions on the adsorption of proteins/peptides onto the carbon-based nanomaterials^30^,^62^,^63^,^64^,^65^,^66^,^67^,^68^,^69^,^70^,^71^,^72^.

The above discussions clearly demonstrates that the vdW dispersion interactions are the common driving force for the absorption of Arg-391 and Tyr-19 in BFG onto the graphene surface, despite the difference in the physicochemical properties of their side chains. As mentioned above, in order to further confirm this important role of basic residues on the adsorption of blood proteins onto graphene surface, we carried out additional simulations (a set of 3 × 200 ns) for the adsorption of BSA onto graphene. As shown in Figure S3, the basic residues (blue surfaces) are again found to play an important role along with aromatic residues during the BSA adsorption process, consistent with that in BFG.

Furthermore, as shown in both Fig. S2 (BFG) and Fig. S3 (BSA), these basic residues (colored in blue) are largely randomly distributed over the protein surfaces in both protein systems. For example, BFG displays a rather uniform distribution of basic residues (aromatic residues too) in both orientations studied (system-1 and system-2; Fig. S2), with the number of basic residues (system-1, 33; system-2, 32) roughly equal to the number of acidic residues (system-1, 34; system-2, 36), but slightly more than the aromatic residues (system-1, 18; system-2, 24). From the analysis of contact probability and interaction energy above, we can clearly see that the basic and aromatic residues are more important than the other types of residues. More interestingly, the contacts and interaction energies displayed a “burst-like” pattern (Fig. 2) rather than a “smoothly-increasing” pattern, and most of these “bursts” were ignited by the binding of basic and/or aromatic residues onto the surface of graphene. During this adsorption process, these long and flexible *α*-helix tails of BFG underwent large conformational changes, with some basic and aromatic residues that were originally in the interior now exposed to the graphene surface.

Finally, it should be noted that the π-π interactions essentially are the interactions between π-electron systems (interaction between the parallel π-systems is specially termed as “π-π stacking interaction”). The negatively charged and diffuse electron clouds of the π-systems exhibit an attractive interaction due to the London dispersion forces which are caused by favorable instantaneous multipole/induced multipole charge fluctuations. However, in molecular mechanics (MM) calculations of aromatic molecule and graphene interactions using modern classical force fields, the graphene carbon atoms are usually modeled as uncharged Lenard-Jones particles, thus the π-π stacking interactions simply reduce to vdW terms solely. Although not explicitly treated, the contribution of electrostatic interactions is well compensated by the vdW terms in these force fields. Our recent work, which combined both quantum mechanical-based dispersion corrected DFTB-D method and classical molecular mechanics-based MD simulations, has demonstrated that classical force fields can properly model the energetic strength of π-π stacking interaction between aromatic amino acid analogues and carbon-based nanomaterials^70^.

## Conclusions

Recently, graphene has attracted increased attention in the biomedical field. Because of its unique electrical, mechanical and chemical properties, graphene has been applied to different drug delivery platforms or even as nanodrugs in both diagnostic and therapeutic endeavors. However, the detailed mechanisms about how graphene interacts with biomolecules, such as lipids, proteins, and nucleic acids, crucial for the study of the biocompatibility and cytotoxicity of graphene, are still not well understood^73^,^74^,^75^. Strong evidences indicate that, upon contact with a bloodstream, graphene will quickly adsorb massive blood proteins to form protein graphene corona complex,which has been found to alleviate its cytotoxicity.

In this study, we have investigated the underlying molecular mechanisms of the adsorption of blood proteins BFG and BSA (in particular BFG due to its unique 3D topology) onto the graphene surface through extensive large-scale all-atom MD simulations. We calculated the contact surface areas and the heavy atom contact number between blood proteins and the graphene surface during the adsorption process, and found a successive bursts-like adsorption pattern. The important intermediate states during the adsorption process have been carefully examined and key residues for the adsorption of both BFG and BSA onto the graphene surface have been identified by studying the time evolution of the vdW interaction energy and the contact probability of each residue with graphene.

To our surprise, in addition to aromatic residues, basic residues were also found to play a very important role. For example, in BFG, the dynamic process of a representative aromatic residue, Tyr-18 in the *γ*-chain, and a representative basic residue, Arg-391 in the *γ*-chain, as identified to have played an important role in the adsorption process, has been studied in detail. We found that while the π−π stacking interactions attract Tyr-18 to bind to the graphene surface, the strong vdW dispersion interactions between the long and relatively planar sidechain (guanidinium group) chain and the graphene provides the driving force for the adsorption of Arg-391. Those with equally long side chains and hydrophobic residues such as Leu/Ile were not found to play an important role during the adsorption process, because Leu/Ile are highly packed in the hydrophobic environment of the interior of the protein, while Arg/Lys are usually exposed to solvent in the native structure of the protein. These detailed systematic analyses provided a more complete molecular picture for the protein-graphene corona complex formation.

## Additional Information

**How to cite this article**: Gu, Z. *et al.* The role of basic residues in the adsorption of blood proteins onto the graphene surface. *Sci. Rep.* **5**, 10873; doi: 10.1038/srep10873 (2015).

## Supplementary Material

Supplementary Information
