# Supplementary material for: The role of basic residues in the adsorption of blood proteins onto the graphene surface
Source: Sci Rep. 2015 Jun 2;5:10873. doi: 10.1038/srep10873 (PMC4451687; doi:10.1038/srep10873)
Supplement: Supplementary Information [file srep10873-s1.pdf]

Supporting Information

The role of basic residues in the adsorption of blood proteins onto the graphene surface

Zonglin Gu[1,‡], Zaixing Yang[1,‡], Lingle Wang[1], Hong Zhou[1], Camilo A. Jimenez-Cruz[2], Ruhong Zhou[1,2,3*]

[1] Institute of Quantitative Biology and Medicine, SRMP and RAD-X, Collaborative Innovation Center of Radiation Medicine of Jiangsu Higher Education Institutions, Soochow University, Suzhou 215123, China

[2] IBM Thomas J. Watson Research Center, Yorktown Heights, NY 10598, USA

[3] Department of Chemistry, Columbia University, New York, NY 10027, USA

‡These authors contribute equally

*Corresponding author, E-mail: ruhongz@us.ibm.com

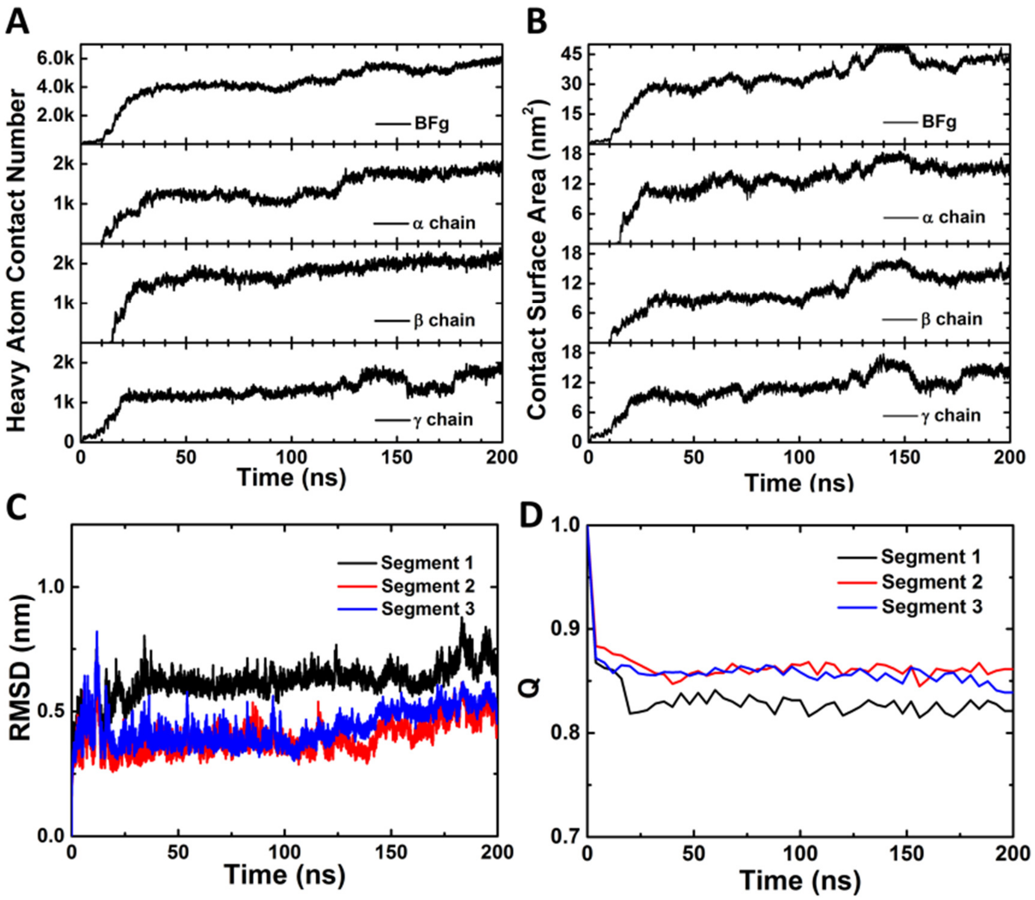

Fig. S1. A representative trajectory of system-2 to shown the adsorbing process of BFG onto the surface of graphene. (A and B, from top to bottom) The heavy atom contact number and contact surface area between GO and BFG, and its branced chains (α-chian, β-chain and γ-chain), respectively. (C and D) RMSD and Q value of α-chian, β-chain and γ-chain versus simulation time, repectively.

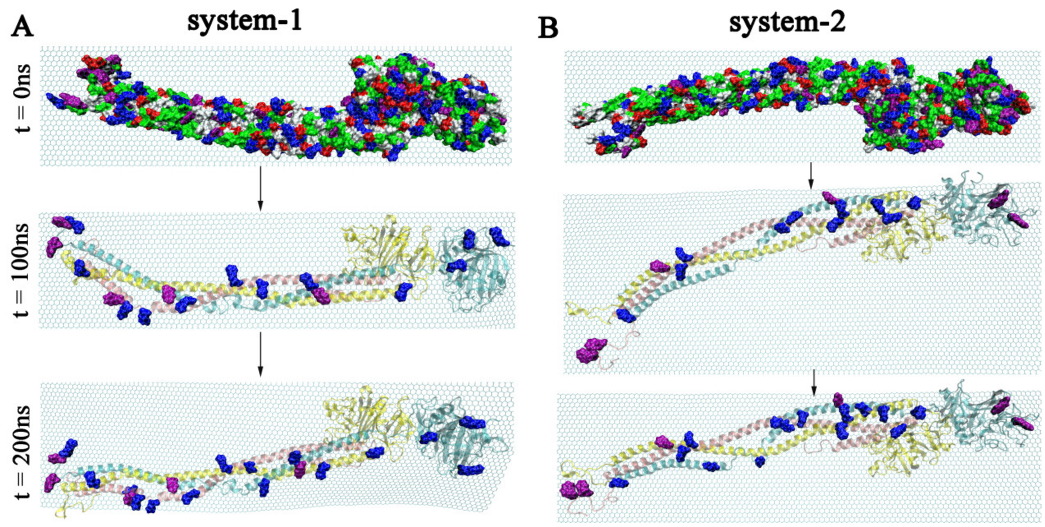

Figure S2. Three representative snapshots at t =0, 100 and 200ns are shown to highlight the graphene-contacting residues of BFG in system-1 (A) and system-2 (B), with the basic, acidic, aromatic, hydrophobic and hydrophilic residues colored in blue, red, purple, white and green surfaces, respectively.

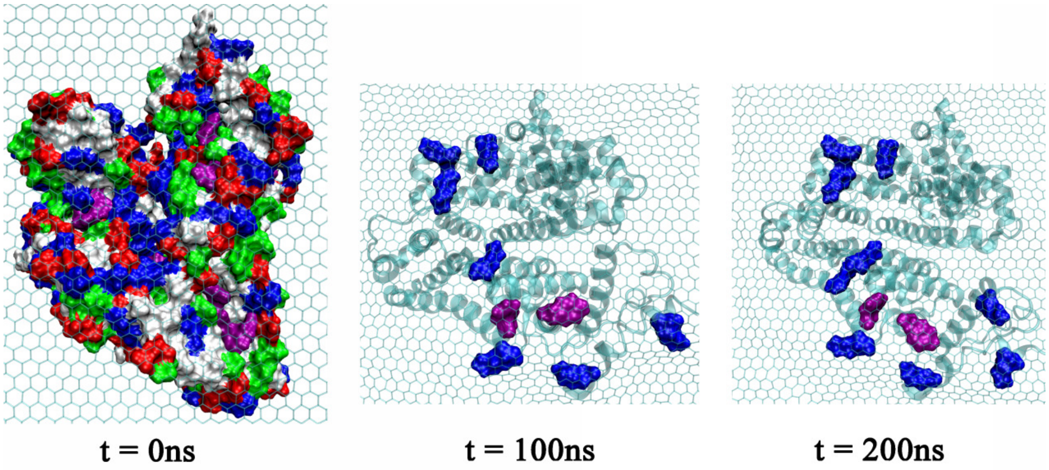

Figure S3. Three representative snapshots at t =0, 100 and 200 ns are shown to highlight the graphene-contacting residues of BSA, with basic, acidic, aromatic, hydrophobic and hydrophilic residues colored in blue, red, purple, white and green surfaces, respectively. In order to further confirm this important role of basic residues on the adsorption of blood proteins onto graphene surface, we carried out another set of 3×200ns additional simulations for the adsorption of BSA (PDB ID: 4F5S)[1] onto graphene (size: 9.8×10.1 nm^2). The simulation box contained 217,519 atoms.

The MD simulation was performed using the software package GROMACS (version 4.6.4) [2] with CHARMM 27 force field[3]. The TIP3P water model[4] was used for the water molecules. As shown in Figure S3, the basic residues (blue surfaces) are again found to play an important role along with aromatic ones during the BSA adsorption process, consistent with that in BFG.

Reference

1 Bujacz, A. Structures of bovine, equine and leporine serum albumin. *Acta Crystallogr. Sect. D-Biol. Crystallogr.* **68**, 1278-1289 (2012).

2 Hess, B., Kutzner, C., van der Spoel, D. & Lindahl, E. GROMACS 4: Algorithms for highly efficient, load-balanced, and scalable molecular simulation. *J. Chem. Theory Comput.* **4**, 435-447 (2008).

3 Mackerell, A. D., Feig, M. & Brooks, C. L. Extending the treatment of backbone energetics in protein force fields: Limitations of gas-phase quantum mechanics in reproducing protein conformational distributions in molecular dynamics simulations. *J. Comput. Chem.* **25**, 1400-1415 (2004).

4 Jorgensen, W. L., Chandrasekhar, J., Madura, J. D., Impey, R. W. & Klein, M. L. Comparison of simple potential functions for simulating liquid water. *J. Chem. Phys.* **79**, 926-935 (1983).
